# Retirement age does not modify the association of prior working conditions with self-rated health and mortality in retirees: results from a prospective study of retired French workers

**DOI:** 10.1007/s00420-022-01886-0

**Published:** 2022-06-10

**Authors:** Nicolas Hoertel, Marina Sanchez Rico, Frédéric Limosin, Cédric Lemogne, Jesús M. Alvarado, Marcel Goldberg, Marie Zins, Joël Ménard, Pierre Meneton

**Affiliations:** 1 Département de Psychiatrie, AP-HP, Hôpital Corentin-Celton, Université de Paris, Issy-les-Moulineaux, France; 2 UMR_S1266, INSERM, Université de Paris, Institut de Psychiatrie et Neurosciences de Paris, Paris, France; 3 Faculté de Médecine, Université de Paris, Paris, France; 4 Department of Psychobiology and Behavioral Sciences Methods, Faculty of Psychology, Universidad Complutense de Madrid, Campus de Somosaguas, Pozuelo de Alarcon, Spain; 5 UMS_011, INSERM, Université Paris-Saclay, Villejuif, France; 6 UMR_1142, INSERM, Sorbonne, Université, Université Paris 13, Paris, France

**Keywords:** Working conditions, Retirement age, Self-rated health, Mortality

## Abstract

**Objective:**

It is unclear whether retirement age can modify the association of working conditions with health and mortality in retirees who are no longer exposed to these conditions.

**Methods:**

The present study investigated this issue in a cohort of 13,378 French workers in whom self-rated health and mortality were measured over 15 years after statutory retirement. The analyses were also performed in homogenous clusters of workers differentiated on the basis of working conditions, social position, birth and retirement years.

**Results:**

Bad working conditions before retirement, which were assessed using a global score combining 25 different occupational exposures, were associated with higher rates of suboptimum self-rated health and mortality in retirees after adjusting for retirement age, social position, demographics and health status before retirement. These rates were also substantially higher in the cluster of workers characterized by bad working conditions in comparison to other clusters. In contrast, retirement age was not associated with self-rated health or mortality after adjusting for working conditions, social position, demographics and health status before retirement. Likewise, no association of retirement age with self-rated health or mortality was found in any cluster of workers and no interactions were observed with any of these clusters.

**Conclusion:**

These results suggest that bad working conditions before retirement have long-term detrimental effects on health and mortality in retirees and that retirement age does not modulate these effects. Improving work environment rather than modifying retirement age should be prioritized to promote health and reduce mortality not only in workers but also in retirees.

**Supplementary Information:**

The online version contains supplementary material available at 10.1007/s00420-022-01886-0.

## Introduction

The conditions in which people work during their lifetime have a profound influence on their health (Burgard and Lin 2013). For example, workers exposed to high demands combined with a low level of control over their job, a situation called job strain, have a shorter health expectancy (Magnusson Hanson et al. 2018). Their mortality risk is increased by 20% over an average period of 14 years (Amiri and Behnezhad 2020), an increase that reaches 60% in workers with cardiometabolic disease (Kivimäki et al. 2018). Likewise, workers exposed to physically demanding jobs have shorter health and life expectancies (Platts et al. 2017). The mechanisms by which bad working conditions can increase mortality and negatively impact health are still debated but they may involve higher risk of musculoskeletal problems (Lang et al. 2012), psychiatric disorders (Theorell et al. 2015), cardiovascular events (Kivimäki and Kawachi 2015) and associated conditions such as obesity, diabetes, dyslipidemia and hypertension (Meneton et al. 2018).

The question arises whether retiring from an adverse work environment, preferably earlier, can bring some positive effects on health and reduce mortality (Baker et al. 1982; Myers 1954). Several studies suggest that retirement is associated with an improvement of mental health but the evidence on its effect on perceived general health and physical health is contradictory (van der Heide et al. 2013). Beneficial health effects of exit from work are mainly observed among retirees with a high socioeconomic status, particularly in the case of early statutory retirement, and much less in those with a low status (Schaap et al. 2018). The evidence for an association between retirement age and mortality is also mixed (Shim et al. 2013), although studies that used adequate reference groups (i.e., working and not general population groups) and adjusted for health and demographic factors generally concluded to the absence of association between retirement age and mortality (Sewdas et al. 2020). However, the difficulty of interpreting these findings is that working conditions before retirement are usually not taken into account (Imamura et al. 2019). The issue is similar for people who lose their job, most studies suggest that unemployment is associated with adverse health effects and increased mortality (Brand 2015; Roelfs et al. 2011), but without considering the potential confounding role of prior working conditions.

Clarifying the relationships between retirement, health and mortality is particularly important at a time where many governments in Europe and elsewhere raise statutory retirement age (OECD 2017a; OECD 2017b). Indeed, among the reasons advanced to justify this policy, the argument that increasing retirement age is necessary because of population aging can be criticized given that life expectancy varies considerably according to work environment and social position. Furthermore, this argument may not necessarily hold for healthy life expectancy that does not rise to the same extent than life expectancy and even reaches a ceiling in some countries (GBD DALYs and HALE Collaborators 2018).

The present study was performed in a well-characterized cohort of French retired workers in whom mortality and self-rated health, which is a strong predictor of mortality (DeSalvo et al. 2006), were measured over a period of more than 15 years after retirement. Our aims were twofold: first, we sought to examine to which extent working conditions along with social position, demographics and health status before retirement predict self-rated health and mortality of retirees; second, we sought to assess whether retirement age is associated with self-rated health and mortality after retirement in homogenous clusters of workers differentiated on the basis of working conditions, social position, demographics and health status before retirement. The use of cluster analysis allowed to group workers in such a way that those included in the same group were more similar to each other than to those included in a different group (Mount and Zumel 2019). This approach has two main advantages: first, it is possible to represent univariate survival curves while still minimizing the potential confounding bias; second, the number of stratifications is reduced, thus limiting the risk of type 1 error (Kassambara 2017).

## Methods

### Study population

The GAZEL cohort was established in 1989 among employees of the French national gas and electricity company, Electricité de France-Gaz de France (EDF-GDF) (Goldberg et al. 2007). At baseline, 20,625 employees (15,011 (73%) men), aged 35–50 years, gave written consent to participate and provide information about their health, lifestyle and socio-occupational status through yearly surveys. EDF-GDF employees had civil-servant-like status that entailed job security and opportunities to move up the career ladder. Typically, employees were hired when they were in their 20s and stayed in the company until retirement after an average career length of 32.6 (SD=4.5) years (Westerlund et al. 2009). Due to the characteristics of the recruitment in the company, the social gradient in the cohort was reduced compared to that of a nationally representative sample of workers, with an overrepresentation of secondary educational level and intermediate occupational grade along with an underrepresentation of primary educational level and blue collar/clerk occupational grade (Table S1). Cohort participants were also healthier as estimated by the lower prevalence of obesity, suboptimum self-rated health and unhealthy lifestyles such as physical inactivity and smoking (Table S1). The study received approval from both the Ethics Evaluation Committee of the French National Institute of Health and Medical Research and the National Committee for the Protection of Privacy and Civil Liberties.

In the present report, we analyzed data from GAZEL workers who retired between 1991 and 2003 and were followed until January 2015 for mortality and January 2013 for self-rated health such that we had for each worker yearly data for at least 2 years before retirement and during a median follow-up period of 16.0 (SD=3.7) years for mortality and 21.0 (SD=4.7) years for self-rated health after retirement. Workers who retired earlier on health grounds, i.e., with longstanding illness or disability, or permanent sickness absence defined as having more than 650 days of sickness absence in the two consecutive years preceding retirement, were excluded as well as individuals lost to follow-up (Fig S1). The final sample retained for the analyses consisted of 13,378 workers (10,803 (80.8%) men) whose baseline characteristics were very similar to those of the whole cohort (Table S1).

### Retirement age

The age of retirement ranged from 37.4 to 63.0 years with a median of 54.3 (SD=2.1) years (Fig. S2). Half of the workers (50.3%) retired on time but early retirement was possible, mainly due to general social agreement (47.2% of workers) and marginally to spouse retirement (people who chose to retire when their spouse retired, 1.5% of workers) or multiple motherhood (women allowed to retire earlier if they had at least three children, 1.0% of workers). Generally, statutory retirement age (retirement on time and early retirement due to general social agreement) was inversely correlated to working conditions: the worse were working conditions (mainly in manual-labor or unskilled roles), the earlier workers were allowed to retire (Fig S3). Note that no national reform or company policy change regarding the age of retirement occurred during the 1991-2003 observation period of the present study.

### Data collection

Death dates were assessed from January 1989 to January 2015 using the linked administrative database CépiDc (Centre d'épidémiologie sur les causes médicales de décès) managed by the French National Institute of Health and Medical Research*.* We assessed self-rated health from January 1989 to January 2013 by yearly questionnaires sent to all participants with the following question: “How would you judge the state of your general health?” The participants responded on an 8-point Likert scale (1=very good, 8=very poor), which was dichotomized by categorizing response scores 1–4 as good health and scores 5–8 as suboptimum health, as previously validated (Niedhammer and Chea 2003).

Among demographic factors, we included sex, birth years (categorized into terciles: 1939-1943, 1944-1946, 1947-1954) and retirement years (also categorized into terciles: 1991-1998, 1998-2000, 2001-2003). Social position and work environment were measured at baseline, i.e., 9.5 (SD=3.1) years on average before retirement, using global scores that combined, respectively, four socioeconomic indicators (education, wealth, income, occupational grade) and 25 physical, biomechanical, organizational and psychosocial occupational exposures, as previously described (Meneton et al. 2017). These 2 scores were, respectively, categorized into high, middle or low social position and good, average or bad working conditions, on the basis of prior evidence supporting those categorizations (Meneton et al. 2018). A checklist was used to identify the following physical illnesses: chronic bronchitis or asthma, angina, myocardial infarction, stroke, diabetes, or cancer. An affirmative response for one or more of these illnesses within the 2 years preceding retirement defined the presence of a physical illness. Depression, sleep and musculoskeletal problems and high sickness absence were assessed in the same timeframe. The presence of musculoskeletal problems was defined as an affirmative response to any musculoskeletal illness or complaint (back, neck or shoulder pain; arthritis; rheumatoid arthritis; sciatica). High sickness absence, obtained from company records, was defined as more than 21 consecutive sick-leave days. Unhealthy behaviors such as smoking, non-moderate alcohol consumption (<14 or >27 drinks/week in men, <7 or >20 drinks/week in women) and leisure-time physical inactivity defined by lack of sport practice whatever its frequency (occasionally, regularly or competition) were also assessed in the 2 years preceding retirement in a subset of the cohort (*n*=2,017).

## Statistical analysis

Percentages were calculated to provide descriptive information about the relationships of working conditions, social position, demographics (i.e., sex, birth and retirement years), health status before retirement (i.e., history of hospitalization, physical illness, depression, sleep problems, musculoskeletal problems, and high sickness absence) and retirement age (divided into terciles, i.e., earlier 37 to 52, medium 53 to 54, and later 55 to 60 years old) with mortality and self-rated health of retirees. To test these associations, we used multivariable weighted Cox regression models including all these variables to determine unbiased average hazard ratio estimates in case of non-proportional hazards (Dunkler et al. 2018). Further adjustments for smoking, leisure-time physical inactivity and non-moderate alcohol consumption before retirement were performed to ensure that unhealthy behaviors may not have residual confounding effects on potential significant associations.

To examine whether the associations of aforementioned characteristics of workers before retirement with mortality and self-rated health after retirement were only observed, or had a greater magnitude, in specific subpopulations of workers and to limit the number of stratifications to minimize the risk of type 1 errors, we used an exploratory individual-based cluster analysis model to determine homogenous groups based on working conditions, social position, demographics and health status before retirement. The number of clusters retained for the analyses was based on cluster performance and clinical meaningfulness. Cluster performance was evaluated using average Silhouette coefficient (Rousseeuw 1987) that measures how well (or badly) each participant is assigned to each cluster, providing a value between 0 and 1 where a higher value indicates a better fit. The selection of the model was then made according to the highest values of average Silhouette coefficient, while checking clinical meaningfulness of the results. Next, we estimated survival Kaplan–Meier curves and performed univariate Cox proportional hazards models to examine the associations of the clusters of workers with mortality and suboptimum self-rated health in retirees. Finally, to assess the impact of retirement age on these associations, overall survival curves by retirement age were estimated by the Kaplan–Meier method. Weighted Cox regression multivariable models including the interaction term retirement age*cluster were also tested to examine whether the strength of the associations of retirement age with mortality and suboptimum self-rated health in retirees differed across the clusters of workers.

For all significant associations, we performed residual analyses to assess the fit of the data and check assumptions. The absence of multicollinearity was verified using the generalized variance inflation factor (GVIF) (Fox and Monette 1992) for each covariable included in every multivariable model. To examine the potential influence of outliers, we performed a sensitivity analysis by running the main models while excluding extreme Martingale-based residuals, i.e., observations that were more than 2 interquartile ranges below the first quartile or above the third quartile (Karasoy and Tuncer 2015). The proportional hazard assumption was checked using Kaplan–Meier curves and by performing goodness-of-fit tests (Gill and Schumacher 1987). We used weighted Cox regression models when the proportional hazard assumption was not met (Dunkler et al. 2018). E-values were used to quantify sensitivity of the findings to unmeasured confounders in the main analyses (Haneuse et al. 2019). Since our approach was both semi-confirmatory and semi-exploratory, and to limit type I error inflation, statistical significance was evaluated using a two-sided design with alpha set a priori at 0.01. All analyses were conducted in R software version 3.6.2.

## Results

Most of the 13,378 workers were men and more than a third had bad working conditions (Table S2). Within the 2 years before retirement, 8.0% had physical illness and 23.1% had high sickness absence. During the follow-up period after retirement, 8.2% died and 47.6% reported suboptimum health (Table S2).

**Fig. 1:**
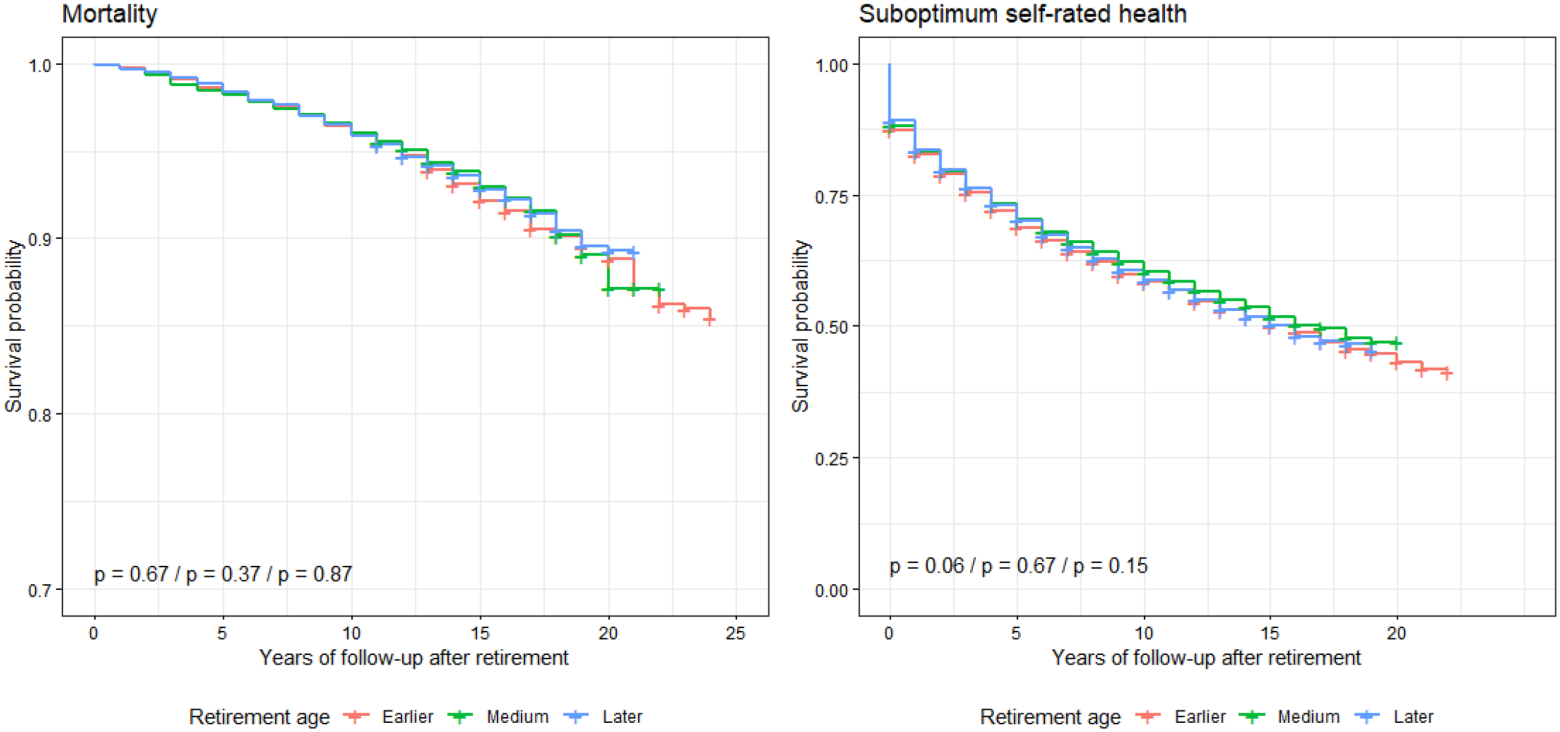
Kaplan–Meier survival curves for mortality and suboptimum self-rated health in retirees by retirement age (*n*=13,378). Log-rank tests *p* values correspond to the comparisons earlier vs. medium retirement age, earlier vs. later retirement age, and medium vs. later retirement age, respectively. Significance was considered met when *p* value <0.01.

Working conditions before retirement were strongly associated with mortality and self-rated health of retirees in univariate analyses (Table S3). Demographics, social position and health status before retirement, but not retirement age, were also associated to a lesser extent with either mortality or self-rated health of retirees (Table S3). When adjusting for all variables, bad working conditions, male sex, and physical illness before retirement were associated with higher mortality, while bad working conditions, low social position, male sex, older age, physical illness, high sickness absence, depression, musculoskeletal and sleep problems before retirement were associated with suboptimum self-rated health (Table 1). As in the univariate analyses, retirement age was not associated with mortality or self-rated health in retirees, which was also supported by Kaplan–Meier survival curves (Fig. 1). The previous results, i.e., bad working conditions before retirement independently associated with increased rates of mortality and suboptimum self-rated health in retirees and retirement age not associated with these two outcomes, held after further adjustment for smoking, leisure-time physical inactivity and non-moderate alcohol consumption before retirement although the statistical significance was *p*<0.05 (and not *p*<0.01 as fixed for the other analyses) because of the limited number of workers (*n*=2,017) for whom unhealthy behaviors were assessed (Table S4).

**Table 1:** Multivariable weighted Cox regression models examining the associations of working conditions, demographics and health status before retirement with mortality and suboptimum self-rated health after retirement (*n*=13,378)

		Mortality				Suboptimum self-rated health			
		HR (SE) ^α^/*p* value	PH assumption (*χ*^2^/*p* value)	GVIF	E-value (lower estimate)	HR (SE) ^β^/*p* value	PH assumption (*χ*^2^/*p* value)	GVIF	E-value (lower estimate)
Working conditions	Good	1.00	7.37/0.0251	1.04		1.00	3.67/0.1598	1.03	
	Average	1.44 (0.16)/0.0216			–	1.20 (0.04) <0.0001^a^			1.53 (1.35)
	Bad	1.43 (0.13)/0.0052^a^			1.86 (1.37)	1.44 (0.04)/<0.0001^a^			1.90 (1.75)
Retirement age	Earlier (37 to 52y)	1.00	0.97/0.6131	1.12		1.00	5.29/0.0711	1.14	
	Medium (53 to 54y)	1.01 (0.11)/0.8901			–	0.94 (0.03)/0.0579			–
	Later (55 to 60y)	0.87 (0.12)/0.2219			–	0.98 (0.04)/0.6447			–
Sex	Women	1.00	1.05/0.3045	1.09		1.00	3.65/0.0559	1.13	
	Men	1.65 (0.18)/0.0067^a^			2.17 (1.43)	0.90 (0.04)/0.0093^a^			1.36 (1.15)
Birth year	1947 to 1954	1.00	0.83/0.6590	1.30		1.00	4.67/0.0968	1.32	
	1944 to 1946	1.04 (0.13)/0.7424			–	1.14 (0.04)/0.0020^a^			1.41 (1.22)
	1939 to 1943	1.46 (0.16)/0.0154			–	1.23 (0.05)/0.0001^a^			1.57 (1.35)
Retirement year	2001 to 2003	1.00	3.14/0.2081	1.27		1.00	13.86/0.0009^a^	1.29	
	1999 to 2000	1.08 (0.12)/0.5365			–	0.98 (0.04)/0.5934			–
	1991 to 1998	0.97 (0.15)/0.8323			–	1.03 (0.05)/0.5908			–
Social position	High	1.00	4.97/0.0832	1.04		1.00	4.35/0.1138	1.03	
	Middle	1.17 (0.14)/0.2437			–	1.06 (0.04)/0.1746			–
	Low	1.22 (0.15)/0.1986			–	1.17 (0.05)/0.0013^a^			1.48 (1.26)
Hospitalization^¥^	No	1.00	9.19/0.0024	1.10		1.00	19.26/<0.0001^a^	1.08	
	Yes	1.00 (0.15)/0.9732			–	1.10 (0.04)/0.0194			–
Physical illness^¥, µ^	No	1.00	6.10/0.0135	1.04		1.00	18.49/<0.0001^a^	1.02	
	Yes	1.77 (0.13)/<0.0001^a^			2.34 (1.79)	1.57 (0.04)/<0.0001^a^			2.07 (1.89)
High sickness absence^¥^	No	1.00	2.10/0.1473	1.14		1.00	34.69/<0.0001^a^	1.11	
	Yes	1.22 (0.13)/0.1322			–	1.33 (0.03)/<0.0001^a^			1.73 (1.60)
Depression^¥^	No	1.00	4.78/0.0287	1.12		1.00	67.09/<0.0001^a^	1.08	
	Yes	0.92 (0.12)/0.4977			–	1.84 (0.03)/<0.0001^a^			2.41 (2.27)
Musculoskeletal problems^¥^	No	1.00	0.89/0.3463	1.09		1.00	44.87/<0.0001^a^	1.07	
	Yes	0.82 (0.14)/0.1539			–	1.26 (0.03)/<0.0001^a^			1.63 (1.51)
Sleep problems^¥^	No	1.00	1.70/0.1925	1.16		1.00	45.59/<0.0001^a^	1.12	
	Yes	0.95 (0.13)/0.6774			–	1.34 (0.03)/<0.0001^a^			1.74 (1.61)

*GVIF* generalized variance inflation factor

^α^Hazard ratios (*HR*) and their standard errors (*SE*) were estimated using multivariable weighted Cox regression models. Proportional hazard assumption *χ*^2^=37.265 (df=7; *p*=0.003)

^β^Hazard ratios (*HR*) and their standard errors (*SE*) were estimated using multivariable weighted Cox regression models. Proportional hazard assumption *χ*^2^=178.62 (df=7; *p*<0.0001)

^**¥**^Assessed during the 2 years before retirement

^**µ**^Chronic bronchitis or asthma, angina, myocardial infarction, stroke, diabetes, or cancer

^a^*p* values are significant (*p*<0.01)

To determine homogenous groups of workers based on working conditions, social position, demographics and health status before retirement, we used the average silhouette coefficient to select the best-suited clustering model and obtained the maximum value for 8 clusters of workers (Fig S4). This result was easy to interpret with clusters distinguishing from each other mainly by working conditions, social position, birth and retirement years: younger workers who retired between 2001 and 2003 (cluster 1), older workers with bad working conditions who retired between 1991 and 1998 (cluster 2), younger retired workers with high social position (cluster 3), older retired workers with high social position (cluster 4), older retired workers with middle social position (cluster 5), younger retired workers with middle social position (cluster 6), retired workers with low social position (cluster 7), and younger workers who retired between 1999 and 2000 (cluster 8) (Table 2).

**Table 2:** Distribution of working conditions, demographics and health status before retirement in each cluster of workers

		Cluster 1	Cluster 2	Cluster 3	Cluster 4	Cluster 5	Cluster 6	Cluster 7	Cluster 8
		*n*=2839	*n*=1541	*n*=842	*n*=1186	*n*=2138	*n*=2440	*n*=1450	*n*=942
Working conditions	Good	737 (26%)	0 (0%)	342 (40.6%)	724 (61%)	970 (45.4%)	599 (24.5%)	386 (26.6%)	225 (23.9%)
	Average	936 (33%)	0 (0%)	276 (32.8%)	459 (38.7%)	1056 (49.4%)	882 (36.1%)	616 (42.5%)	327 (34.7%)
	Bad	1166 (41.1%)	**1541 (100%)**	224 (26.6%)	3 (0.3%)	111 (5.2%)	959 (39.3%)	447 (30.8%)	390 (41.4%)
Retirement age	Earlier (37 to 52 y)	1072 (37.8%)	425 (27.6%)	124 (14.7%)	139 (11.7%)	477 (22.3%)	782 (32%)	429 (29.6%)	**909 (96.5%)**
	Medium (53 to 54 y)	1233 (43.4%)	582 (37.8%)	259 (30.8%)	204 (17.2%)	654 (30.6%)	990 (40.6%)	468 (32.3%)	33 (3.5%)
	Later (55 to 60 y)	534 (18.8%)	534 (34.7%)	459 (54.5%)	843 (71.1%)	1007 (47.1%)	668 (27.4%)	553 (38.1%)	0 (0%)
Sex	Women	774 (27.3%)	114 (7.4%)	92 (10.9%)	128 (10.8%)	475 (22.2%)	422 (17.3%)	374 (25.8%)	196 (20.8%)
	Men	2065 (72.7%)	1427 (92.6%)	750 (89.1%)	1058 (89.2%)	1663 (77.8%)	2018 (82.7%)	1076 (74.2%)	746 (79.2%)
Birth year	1947 to 1954	**2837 (99.9%)**	2 (0.1%)	1 (0.1%)	13 (1.1%)	4 (0.2%)	0 (0%)	4 (0.3%)	**942 (100%)**
	1944 to 1946	2 (0.1%)	0 (0%)	**839 (99.6%)**	10 (0.8%)	0 (0%)	**2440 (100%)**	734 (50.6%)	0 (0%)
	1939 to 1943	0 (0%)	**1539 (99.9%)**	2 (0.2%)	**1163 (98.1%)**	**2134 (99.8%)**	0 (0%)	712 (49.1%)	0 (0%)
Retirement year	2001 to 2003	**2668 (94%)**	34 (2.2%)	371 (44.1%)	202 (17%)	128 (6%)	491 (20.1%)	215 (14.8%)	0 (0%)
	1999 to 2000	0 (0%)	46 (3%)	319 (37.9%)	310 (26.1%)	250 (11.7%)	1114 (45.7%)	430 (29.7%)	**942 (100%)**
	1991 to 1998	171 (6%)	**1461 (94.8%)**	152 (18.1%)	674 (56.8%)	1760 (82.3%)	835 (34.2%)	805 (55.5%)	0 (0%)
Social position	High	530 (18.7%)	264 (17.1%)	**842 (100%)**	**1186 (100%)**	0 (0%)	0 (0%)	2 (0.1%)	114 (12.1%)
	Middle	1703 (60%)	878 (57%)	0 (0%)	0 (0%)	**2137 (100%)**	**2435 (99.8%)**	1 (0.1%)	644 (68.4%)
	Low	606 (21.3%)	399 (25.9%)	0 (0%)	0 (0%)	1 (0%)	5 (0.2%)	**1447 (99.8%)**	184 (19.5%)
Hospitalization^¥^	No	2384 (84%)	1312 (85.1%)	714 (84.8%)	987 (83.2%)	1818 (85%)	2045 (83.8%)	1230 (84.8%)	796 (84.5%)
	Yes	455 (16%)	229 (14.9%)	128 (15.2%)	199 (16.8%)	320 (15%)	395 (16.2%)	220 (15.2%)	146 (15.5%)
Physical illness^¥,µ^	No	2617 (92.2%)	1421 (92.2%)	770 (91.4%)	1089 (91.8%)	2000 (93.5%)	2238 (91.7%)	1309 (90.3%)	866 (91.9%)
	Yes	222 (7.8%)	120 (7.8%)	72 (8.6%)	97 (8.2%)	138 (6.5%)	202 (8.3%)	141 (9.7%)	76 (8.1%)
High sickness absence^¥^	No	2229 (78.5%)	1094 (71%)	731 (86.8%)	1041 (87.8%)	1650 (77.2%)	1879 (77%)	959 (66.1%)	711 (75.5%)
	Yes	610 (21.5%)	447 (29%)	111 (13.2%)	145 (12.2%)	488 (22.8%)	561 (23%)	491 (33.9%)	231 (24.5%)
Depression^¥^	No	2345 (82.6%)	1219 (79.1%)	706 (83.8%)	1024 (86.3%)	1855 (86.8%)	2000 (82%)	1166 (80.4%)	728 (77.3%)
	Yes	494 (17.4%)	322 (20.9%)	136 (16.2%)	162 (13.7%)	283 (13.2%)	440 (18%)	284 (19.6%)	214 (22.7%)
Musculoskeletal problems^¥^	No	1429 (50.3%)	584 (37.9%)	434 (51.5%)	631 (53.2%)	942 (44.1%)	1116 (45.7%)	674 (46.5%)	435 (46.2%)
	Yes	1410 (49.7%)	957 (62.1%)	408 (48.5%)	555 (46.8%)	1196 (55.9%)	1324 (54.3%)	776 (53.5%)	507 (53.8%)
Sleep problems^¥^	No	2127 (74.9%)	998 (64.8%)	646 (76.7%)	898 (75.7%)	1544 (72.2%)	1730 (70.9%)	1034 (71.3%)	666 (70.7%)
	Yes	712 (25.1%)	543 (35.2%)	196 (23.3%)	288 (24.3%)	594 (27.8%)	710 (29.1%)	416 (28.7%)	276 (29.3%)

Cluster 1 younger workers who retired between 2001 and 2003, Cluster 2 older workers with bad working conditions who retired between 1991 and 1998, Cluster 3 younger retired workers with high social position, Cluster 4 older retired workers with high social position, Cluster 5 older retired workers with middle social position, Cluster 6 younger retired workers with middle social position, Cluster 7 retired workers with low social position, Cluster 8 younger workers who retired between 1999 and 2000

Values in bold indicate that nearly all participants from the cluster have the corresponding characteristics

**Fig. 2:**
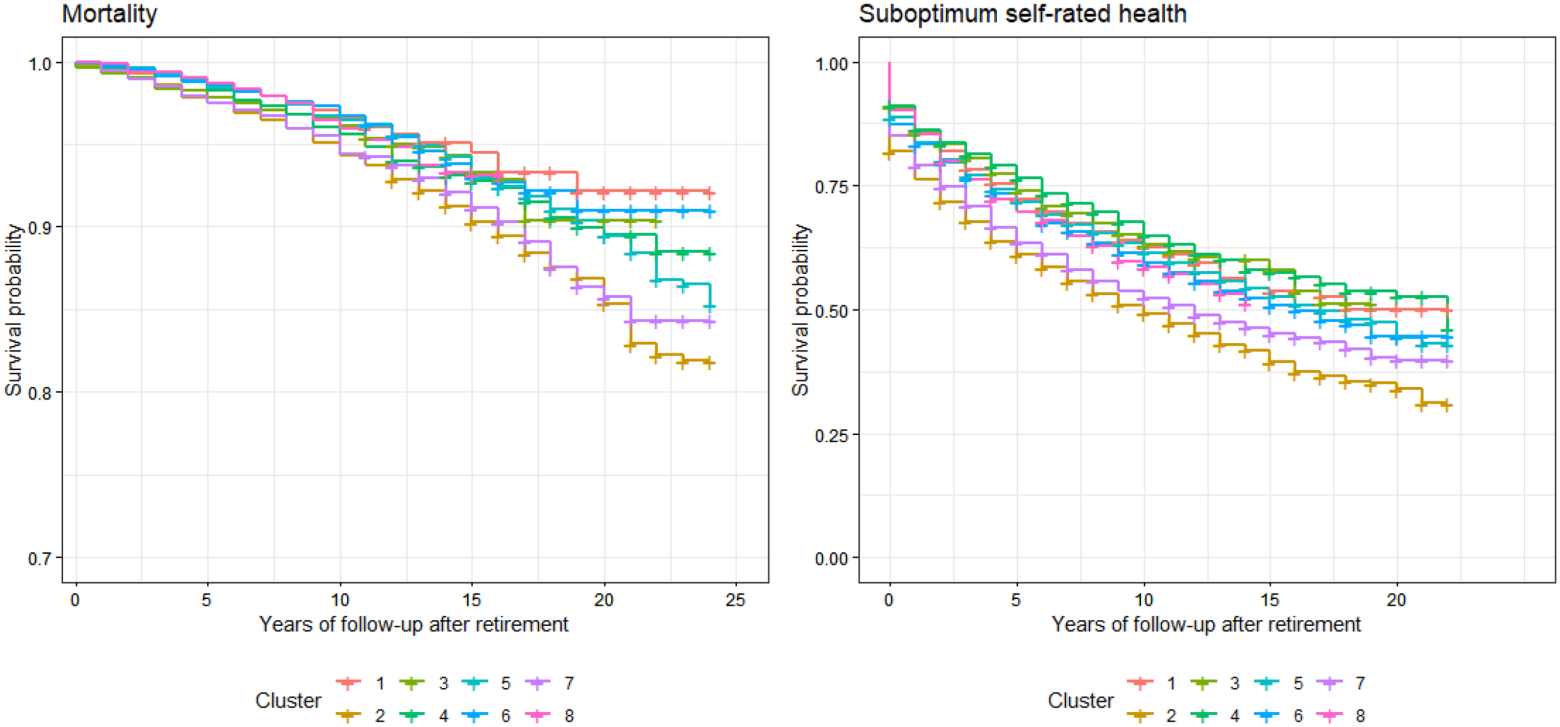
Kaplan–Meier survival curves for mortality and suboptimum self-rated health in retirees by cluster of workers. Cluster 1 younger workers who retired between 2001 and 2003, Cluster 2 older workers with bad working conditions who retired between 1991 and 1998, Cluster 3 younger retired workers with high social position, Cluster 4 older retired workers with high social position, Cluster 5 older retired workers with middle social position, Cluster 6 younger retired workers with middle social position, Cluster 7 retired workers with low social position, Cluster 8 younger workers who retired between 1999 and 2000

**Fig. 3:**
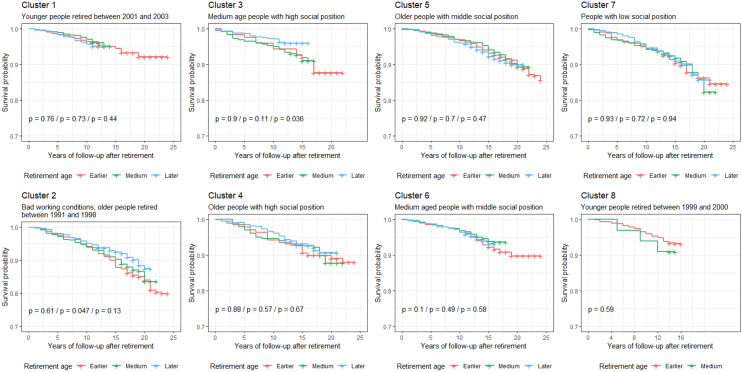
Kaplan–Meier survival curves examining the influence of retirement age on mortality in retirees by cluster of workers. Log-rank tests *p* values correspond to the comparisons earlier vs. medium retirement age, earlier vs. later retirement age, and medium vs. later retirement age, respectively. Significance was considered met when *p* value <0.01

**Fig. 4:**
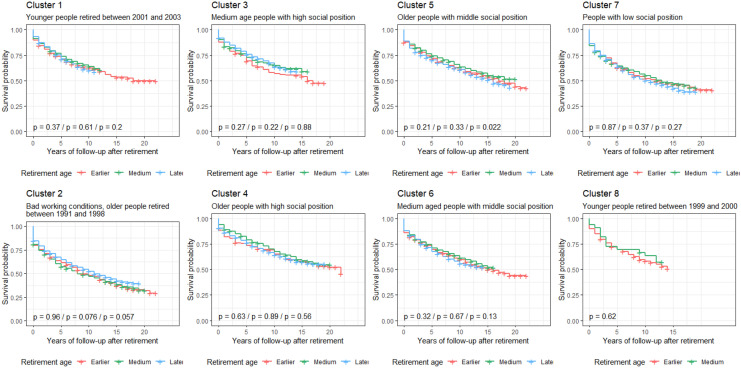
Kaplan–Meier survival curves examining the influence of retirement age on suboptimum self-rated health in retirees by cluster of workers. Log-rank tests *p* values correspond to the comparisons earlier vs. medium retirement age, earlier vs. later retirement age, and medium vs. later retirement age, respectively. Significance was considered met when *p* value <0.01

Kaplan–Meier curves (Fig. 2) and univariate Cox regression models (Table 3) supported substantial differences across clusters of workers in rates of mortality and suboptimum self-rated health after retirement. These associations were maintained when excluding outliers (Table S5). Older retired workers with bad working conditions (cluster 2) had higher rates of mortality and suboptimum self-rated health than older retired workers with high social position (cluster 4). In addition, workers with low social position (cluster 7), older and younger workers with middle social position (clusters 6 and 7) and younger workers who retired between 1999 and 2000 (cluster 8) had a higher rate of suboptimum self-rated health than older retired workers with high social position (cluster 4). Rates of mortality and suboptimum self-rated health remained significantly higher (at *p*<0.05) in the cluster of workers characterized by bad working conditions in comparison to other clusters after adjustment for smoking, leisure-time physical inactivity and non-moderate alcohol consumption before retirement (Table S6). Finally, retirement age was not significantly associated with mortality (Fig. 3 and Table S7) or self-rated health (Fig. 4 and Table S7) in any cluster of workers and there was no interaction between retirement age and any of these clusters (Table S8).

**Table 3:** Univariate Cox proportional hazard regression models examining the associations of clusters of workers with mortality and suboptimum self-rated health after retirement (*n*=13,378)

	Mortality		Suboptimum self-rated health	
Cluster	HR (SE) ^α^/*p* value	E-value (lower estimate)	HR (SE) ^β^/*p* value	E-value (lower estimate)
4	1.00	–	1.00	–
1	0.82 (0.13)/0.1398	–	1.09 (0.05)/0.1068	–
2	1.39 (0.12)/0.0062^a^	1.83 (1.34)	1.71 (0.06)/<0.0001^a^	2.25 (2.02)
3	0.94 (0.17)/0.6958	–	1.03 (0.07)/0.6617	–
5	0.98 (0.12)/0.885	–	1.18 (0.05)/0.0025^a^	1.17 (1.00)
6	0.92 (0.13)/0.5363	–	1.23 (0.05)/0.0001^a^	1.48 (1.25)
7	1.29 (0.13)/0.0487	–	1.48 (0.06)/<0.0001^a^	1.58 (1.36)
8	0.98 (0.16)/0.8981	–	1.25 (0.07)/0.0008^a^	1.60 (1.33)

Cluster 1: younger workers who retired between 2001 and 2003; Cluster 2: older workers with bad working conditions who retired between 1991 and 1998; Cluster 3: younger retired workers with high social position; Cluster 4: older retired workers with high social position; Cluster 5: older retired workers with middle social position; Cluster 6: younger retired workers with middle social position; Cluster 7: retired workers with low social position; Cluster 8: younger workers who retired between 1999 and 2000

^α^Hazard ratios (*HR*) and their standard errors (*SE*) were estimated using Cox proportional hazard regression models. Proportional hazard assumption *χ*^2^=6.59 (df=7; *p*=0.47)

^β^Hazard ratios (*HR*) and their standard errors (*SE*) were estimated using Cox proportional hazard regression models. Proportional hazard assumption *χ*^2^=13.5 (df=7; *p*=0.06)

^a^*p* values are significant (*p*<0.01)

## Discussion

We found that bad working conditions prior to retirement are strongly associated with higher rates of suboptimum self-rated health and mortality in retirees over a follow-up period of more than 15 years, after adjustment for confounding factors such as social position, demographics, health status and unhealthy behaviors before retirement. This result is in line with current evidence even though working conditions in our analyses were assessed more comprehensibly than in most studies (Amiri and Behnezhad 2020; Magnusson Hanson et al. 2018). As previously discussed (Meneton et al. 2017; Meneton et al. 2018), combining all available occupational exposures, each representing a different aspect of work environment (physical, biomechanical, organizational, psychosocial), into a global score allows to consider this environment as a whole, which better mimic the reality for workers who are not facing only one or a few exposures. It is noteworthy that working conditions are a major determinant of health and mortality not only in workers but also in retirees who have stopped working for many years (Imamura et al. 2019). We have previously shown that bad working conditions are strongly associated with the risk of depression, sleep complaints and obesity in active workers (Meneton et al. 2017) and retirees (Meneton et al. 2018), suggesting that the health consequences of an adverse work environment can persist after people are no longer exposed to this environment. This is also supported by findings showing that combined biomechanical and psychosocial occupational exposures during working life act as joint predictors of post-retirement functional health (Sabbath et al. 2013).

In contrast to working conditions, we found that retirement age is not associated with self-rated health or mortality of retirees, neither in the whole cohort after adjustment for confounding factors, nor across homogenous groups differing by prior working conditions, social position, birth and retirement years, which is in agreement with available evidence (Sewdas et al. 2020; van der Heide et al. 2013). It must be mentioned that most retirements in the cohort were statutory, including early retirements that were mainly due to general social agreement, and only marginally voluntary in relation to spouse retirement or multiple motherhood.

The main finding of the present study is that prior working conditions predict suboptimum self-rated health and mortality of retirees independently of a broad range of potential confounders and that retirement age does not modify these associations. This finding supports the view that retiring a few years earlier may not fundamentally change health effects of the conditions in which individuals work for several hours every day during a career of more than 30 years. Combined with the fact that retirement age is not associated by itself with self-rated health and mortality of retirees, this observation suggests that modifying statutory retirement age is not a way to equilibrate inequalities in life expectancy and health of retirees in contrast to policies that would improve working conditions. However, decreasing retirement age allows to increase retirement duration in people with hard jobs and associated low life expectancies (Cambois 2004).

The present study does not provide specific indications for improving working conditions because these latter were assessed globally using a score combining 25 physical, biomechanical, organizational and psychosocial exposures (Meneton et al. 2018). The rationale for this approach is that considering all available occupational exposures, each representing a different aspect of work environment, allows to assess this environment as a whole, which is the reality for workers who usually are not facing only one or a few exposures. Even if some exposures such as job strain that have been shown to be associated with a large increase in mortality (Magnusson Hanson et al. 2018; Amiri and Behnezhad 2020; Kivimäki et al. 2018) are obvious targets for preventive interventions, it must be realized that changing only one or a few exposures will not necessarily improve work environment as a whole.

This study has several limitations. A first one is the external validity of the findings that were obtained in a cohort of healthy and somewhat socially privileged civil servants who were not representative of the French working population as acknowledged in “Methods”. A second is that work environment was assessed 9.5 years on average before retirement and not at the time of retirement nor over the entire career which lasted on average 32.6 years. A third is that occupational data as well as health status were self-reported and may, therefore, be relatively imprecise. A fourth is that our results were obtained in a cohort where the vast majority of workers retired on a statutory basis. A fifth is the early average age at which workers retire compared to other countries where retirement occurs several years later. It is difficult to predict how these limitations could impact the results of the study. It cannot be excluded that in a cohort of socially disadvantaged workers who would retire at an older age, assessing working conditions over the entire career or just before retirement with more accurate methods than self-reporting would show that the age of retirement turns out to modulate the association of these conditions with mortality and health in retirees. The results might also differ in a cohort where most workers would retire on a voluntary basis (van der Heide et al. 2013). Finally, despite the prospective design of the analyses and extensive adjustment for health problems and lifestyle risk factors before retirement, reverse causation where poor health status of workers would trigger bad working conditions cannot be ruled out although it seems unlikely. Severe health problems are known to increase the risk of unemployment rather than to deteriorate work environment (Brand 2015). In any case, it must be recalled that observed associations do not necessarily imply causation (Le Strat and Hoertel 2011).

In conclusion, our study suggests that the age of retirement does not influence health and mortality in retirees and does not modulate the effect of prior working conditions, although it allows to adjust retirement duration on job hardness and associated life expectancy. Our results highlight that improving working conditions rather than modifying retirement age would help to promote health and decrease mortality not only in workers but also in retirees.

## Supplementary Information

Below is the link to the electronic supplementary material.

Supplementary file1 (DOCX 540 KB)

## Data Availability

The data underlying the findings of this study are not publicly available for legal reasons related to data privacy protection. The GAZEL (Gaz and Electricité) cohort has a data sharing policy but a legal authorization must first be obtained from the French National Committee for the Protection of Privacy and Civil Liberties. Email address to contact the staff is gazel@inserm.fr.

## References

[CR1] Amiri S, Behnezhad S (2020). Job strain and mortality ratio: a systematic review and meta-analysis of cohort studies. Public Health.

[CR2] Baker D, Packard M, Rader AD, Reno V, Upp M (1982). Mortality and early retirement. Soc Secur Bull.

[CR3] Brand JE (2015). The far-reaching impact of job loss and unemployment. Annu Rev Sociol.

[CR4] Burgard SA, Lin KY (2013). Bad jobs, bad health? How work and working conditions contribute to health disparities. Am Behav Sci.

[CR5] Cambois E (2004). Careers and mortality in France: evidence on how far occupational mobility predicts differentiated risks. Soc Sci Med.

[CR6] DeSalvo KB, Bloser N, Reynolds K, He J, Muntner P (2006). Mortality prediction with a single general self-rated health question. A meta-analysis. J Gen Intern Med.

[CR7] Dunkler D, Ploner M, Schemper M, Heinze G (2018). Weighted cox regression using the R package coxphw. J Stat Softw.

[CR8] Fox J, Monette G (1992). Generalized collinearity diagnostics. J Am Stat Assoc.

[CR9] GBD DALYs and HALE Collaborators (2018) Global, regional, and national disability-adjusted life-years (DALYs) for 359 diseases and injuries and healthy life expectancy (HALE) for 195 countries and territories, 1990–2017: a systematic analysis for the global burden of disease study 2017. Lancet 392:1859–1922 10.1016/S0140-6736(18)32335-3 PMC6252083 30415748

[CR10] Gill R, Schumacher M (1987). A simple test of the proportional hazard assumption. Biometrika.

[CR11] Goldberg M, Leclerc A, Bonenfant S, Chastang JF, Schmaus A, Kaniewski N, Zins M (2007). Cohort profile: the GAZEL cohort study. Int J Epidemiol.

[CR12] Haneuse S, VanderWeele TJ, Arterburn D (2019). Using the E-value to assess the potential effect of unmeasured confounding in observational studies. JAMA.

[CR13] Imamura K, Tsutsumi A, Asai Y, Arima H, Ando E, Inoue A, Inoue R, Iwanaga M, Eguchi H, Otsuka Y, Kobayashi Y, Sakuraya A, Sasaki N, Tsuno K, Hino A, Watanabe K, Shimazu A, Kawakami N (2019). Association between psychosocial factors at work and health outcomes after retirement: a protocol for a systematic review and meta-analysis. BMJ Open.

[CR14] Karasoy D, Tuncer N (2015). Outliers in survival analysis. Alphanumeric J.

[CR15] Kassambara A (2017). Practical guide to cluster analysis in R: Unsupervised machine learning..

[CR16] Kivimäki M, Kawachi I (2015). Work stress as a risk factor for cardiovascular disease. Curr Cardiol Rep.

[CR17] Kivimäki M, Pentti J, Ferrie JE, Batty GD, Nyberg ST, Jokela M, Virtanen M, Alfredsson L, Dragano N, Fransson EI, Goldberg M, Knutsson A, Koskenvuo M, Koskinen A, Kouvonen A, Luukkonen R, Oksanen T, Rugulies R, Siegrist J, Singh-Manoux A, Suominen S, Theorell T, Väänänen A, Vahtera J, Westerholm PJM, Westerlund H, Zins M, Strandberg T, Steptoe A, Deanfield J, IPD-Work C, (2018). Work stress and risk of death in men and women with and without cardiometabolic disease: a multicohort study. Lancet Diabetes Endocrinol.

[CR18] Lang J, Ochsmann E, Kraus T, Lang JW (2012). Psychosocial work stressors as antecedents of musculoskeletal problems: a systematic review and meta-analysis of stability-adjusted longitudinal studies. Soc Sci Med.

[CR19] Le Strat Y, Hoertel N (2011). Correlation is no causation: gymnasium proliferation and the risk of obesity. Addiction.

[CR20] Magnusson Hanson LL, Westerlund H, Chungkham HS, Vahtera J, Rod NH, Alexanderson K, Goldberg M, Kivimäki M, Stenholm S, Platts LG, Zins M, Head J (2018). Job strain and loss of healthy life years between ages 50 and 75 by sex and occupational position: analyses of 64,934 individuals from four prospective cohort studies. Occup Environ Med.

[CR21] Meneton P, Lemogne C, Herquelot E, Bonenfant S, Czernichow S, Ménard J, Goldberg M, Zins M (2017). Primary cardiovascular disease risk factors predicted by poor working conditions in the Gazel cohort. Am J Epidemiol.

[CR22] Meneton P, Hoertel N, Wiernik E, Lemogne C, Ribet C, Bonenfant S, Ménard J, Goldberg M, Zins M (2018). Work environment mediates a large part of social inequalities in the incidence of several common cardiovascular risk factors: findings from the Gazel cohort. Soc Sci Med.

[CR23] Mount J, Zumel N (2019). Practical data science with R.

[CR24] Myers RJ (1954). Factors in interpreting mortality after retirement. J Amer Statist Ass.

[CR25] Niedhammer I, Chea M (2003). Psychosocial factors at work and self reported health: comparative results of cross sectional and prospective analyses of the French GAZEL cohort. Occup Environ Med.

[CR26] OECD (2017a) Aging and Employment Policies - Statistics on average effective age of of labour market exit. https://www.oecd.org/els/emp/average-effective-age-of-labour-market-exit.htm. Accessed 8 June 2022

[CR27] OECD (2017b) Pensions at a glance 2017: OECD and G20 indicators. https://www.oecdilibrary.org/docserver/pension_glance-2017-en.pdf?expires=1654697689&id=id&accname=guest&checksum=499197704315534CD4647C84B111BE0F. Accessed 8 June 2022

[CR28] Platts LG, Head J, Stenholm S, Singh Chungkham H, Goldberg M, Zins M (2017). Physical occupational exposures and health expectancies in a French occupational cohort. Occup Environ Med.

[CR29] Roelfs DJ, Shor E, Davidson KW, Schwartz JE (2011). Losing life and livelihood: a systematic review and meta-analysis of unemployment and all-cause mortality. Soc Sci Med.

[CR30] Rousseeuw PJ (1987). Silhouettes: a graphical aid to the interpretation and validation of cluster analysis. J Comput Appl Math.

[CR31] Sabbath EL, Glymour MM, Descatha A, Leclerc A, Zins M, Goldberg M, Berkman LF (2013). Biomechanical and psychosocial occupational exposures: joint predictors of post-retirement functional health in the French GAZEL cohort. Adv Life Course Res.

[CR32] Schaap R, de Wind A, Coenen P, Proper K, Boot C (2018). The effects of exit from work on health across different socioeconomic groups: a systematic literature review. Soc Sci Med.

[CR33] Sewdas R, de Wind A, Stenholm S, Coenen P, Louwerse I, Boot C, van der Beek A (2020). Association between retirement and mortality: working longer, living longer? A systematic review and meta-analysis. J Epidemiol Community Health.

[CR34] Shim MJ, Gimeno D, Pruitt SL, McLeod CB, Foster MJ, Amick BC (2013). A systematic review of retirement as a risk factor for mortality. Appl Demogr Public Health.

[CR35] Theorell T, Hammarström A, Aronsson G, Träskman Bendz L, Grape T, Hogstedt C, Marteinsdottir I, Skoog I, Hall C (2015). A systematic review including meta-analysis of work environment and depressive symptoms. BMC Public Health.

[CR36] van der Heide I, van Rijn RM, Robroek SJ, Burdorf A, Proper KI (2013). Is retirement good for your health? A systematic review of longitudinal studies. BMC Public Health.

[CR37] Westerlund H, Kivimäki M, Singh-Manoux A, Melchior M, Ferrie JE, Pentti J, Jokela M, Leineweber C, Goldberg M, Zins M, Vahtera J (2009). Self-rated health before and after retirement in France (GAZEL): a cohort study. Lancet.

