# Supplementary material for: Retirement age does not modify the association of prior working conditions with self-rated health and mortality in retirees: results from a prospective study of retired French workers
Source: Int Arch Occup Environ Health. 2022 Jun 10;95(10):1921–34. doi: 10.1007/s00420-022-01886-0 (PMC9652296; doi:10.1007/s00420-022-01886-0)
Supplement: Supplementary file 1 — Supplementary file1 (DOCX 540 KB) [file 420_2022_1886_MOESM1_ESM.docx]

**Table S1.** Demographics and health characteristics of GAZEL participants compared to randomly selected French workers.

|  | **Men** | | | **Women** | | |
| --- | --- | --- | --- | --- | --- | --- |
|  | **All GAZEL**  **participants** | **Selected GAZEL participants** | **Randomly**  **selected workers** | **All GAZEL**  **participants** | **Selected GAZEL participants** | **Randomly**  **selected workers** |
| **N** | 15,011 | 10,803 | 2168 | 5614 | 2575 | 3107 |
| **Age, y (mean ± SD)** | 44.5 ± 2.9 | 44.9 ± 2.8 | 44.8 ± 3.1 | 41.7 ± 4.2 | 43.8 ± 3.6 | 42.4 ± 4.5 |
| **Physical inactivity, % (n)** | 30.8 (3895) | 30.0 (2893) | 52.3 (1133) | 40.5 (1811) | 42.0 (938) | 57.1 (1773) |
| **Current smoking, % (n)** | 31.0 (4632) | 29.1 (3130) | 34.0 (661) | 21.7 (1200) | 16.6 (422) | 30.8 (874) |
| **Obesity, % (n)** | 5.5 (707) | 5.3 (512) | 11.0 (239) | 3.5 (162) | 3.2 (72) | 8.8 (275) |
| **Suboptimum self-rated health, % (n)** | 13.0 (1937) | 11.3 (1211) | 17.4 (377) | 14.4 (798) | 13.2 (335) | 18.6 (577) |
| **Education, % (n)** |  |  |  |  |  |  |
| Primary school | 6.6 (967) | 6.3 (668) | 19.7 (428) | 7.5 (409) | 8.7 (218) | 15.9 (494) |
| Secondary school | 72.4 (10,644) | 75.0 (7962) | 55.1 (1194) | 79.6 (4343) | 81.4 (2039) | 53.3 (1657) |
| University | 21.0 (3094) | 18.7 (1990) | 25.2 (546) | 12.9 (702) | 9.9 (248) | 30.8 (956) |
| **Occupation, % (n)** |  |  |  |  |  |  |
| Blue collar/clerk | 14.3 (2151) | 12.1 (1302) | 50.4 (1093) | 27.3 (1529) | 20.0 (514) | 59.4 (1845) |
| Intermediate | 55.9 (8384) | 58.1 (6268) | 28.5 (618) | 64.8 (3635) | 72.0 (1852) | 27.3 (849) |
| Management | 29.7 (4459) | 29.8 (3222) | 21.1 (457) | 7.9 (442) | 8.0 (207) | 13.3 (413) |

The table shows baseline characteristics of all GAZEL participants (n=20,624), GAZEL participants who were selected for the present analyses (n=13,378) as described in the Methods, and workers (n=5275) in the same age range (35–50 years) randomly selected from a nationally representative survey conducted in 2003 by the French National Institute of Statistics and Economic Studies (Lanoe JL, Makdessi-Raynaud Y. L’état de santé en France en 2003: santé perçue, morbidité déclarée et recours aux soins à travers l’enquête décennale santé. Etudes et Résultats 436:1–12, 2005).

**Fig S1.** Flow chart describing the selection of participants in the study.

44,922 employees of Electricité de France-Gaz de France company invited in 1989

24,298 declined

20,625 volunteered and form the GAZEL cohort

4307 retired after 2003, are still in work, or left both the company and the study before retirement

16,318 retired in 1991–2003

918 participants excluded because he or she retired on health grounds, i.e., with longstanding illness or disability, or permanent sickness absence in the 2 consecutive years preceding retirement

15,400 without recorded health issues

2022 participants lost to follow-up

**13,378** were included in the present analyses

**Fig S2.** Distribution of retirement age (n=13,378).

**
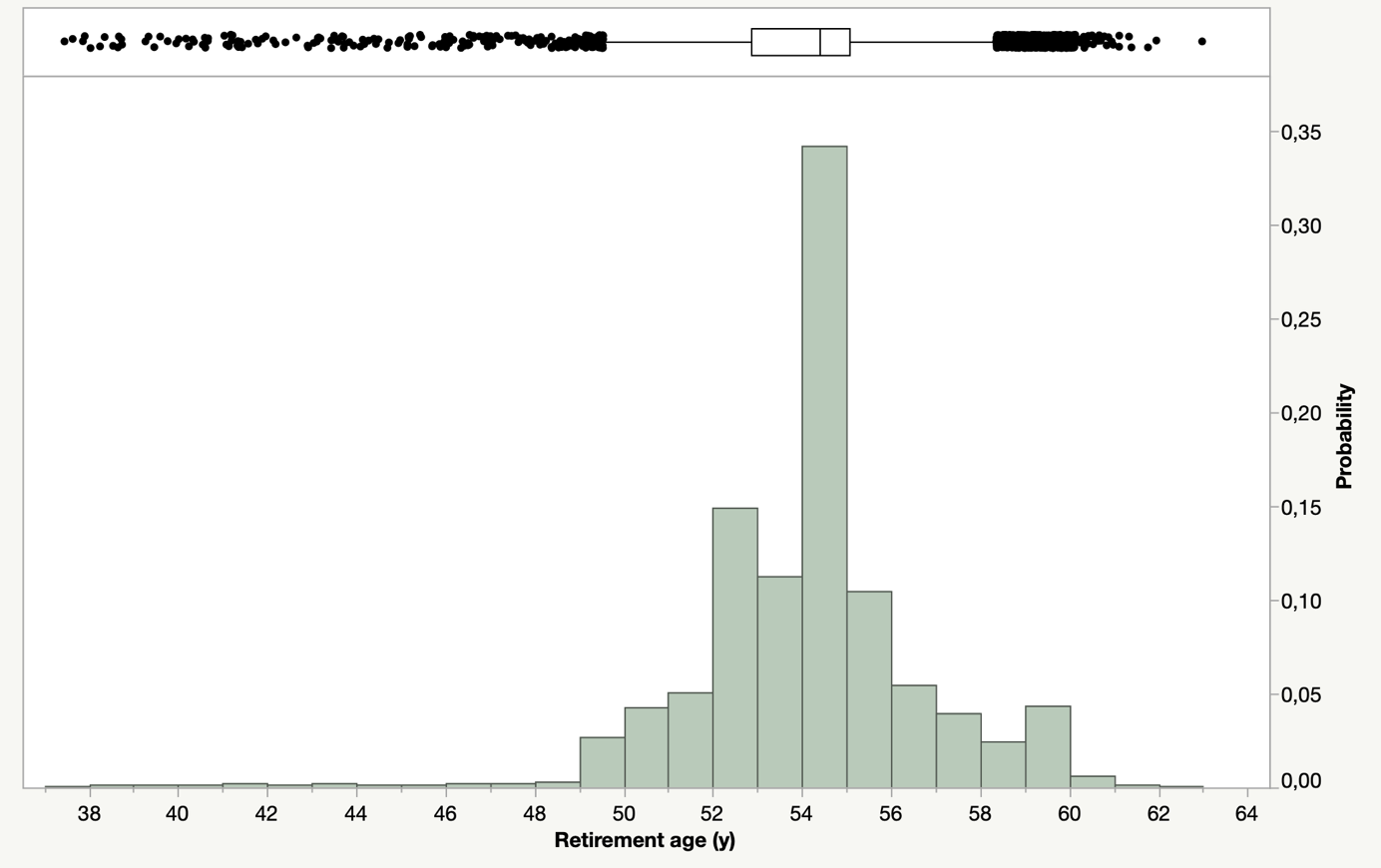
**

In the box plot at the top of the figure, the vertical line represents the median value, the ends of the box represent the 1^st^ and 3^rd^ quartiles and the length of the box is the interquartile range, the lines on each end of the box extend to the outermost values that fall within 1^st^ quartile -1.5*(interquartile range) and 3^rd^ quartile + 1.5*(interquartile range), the values below or above these boundaries are shown as individual outliers.

**Fig S3.** Multiple correspondence analysis showing the association between retirement age and working conditions (n=13,378). The plot uses the two first dimensions which explain respectively 29.0 and 24.9% of the total inertia.

**
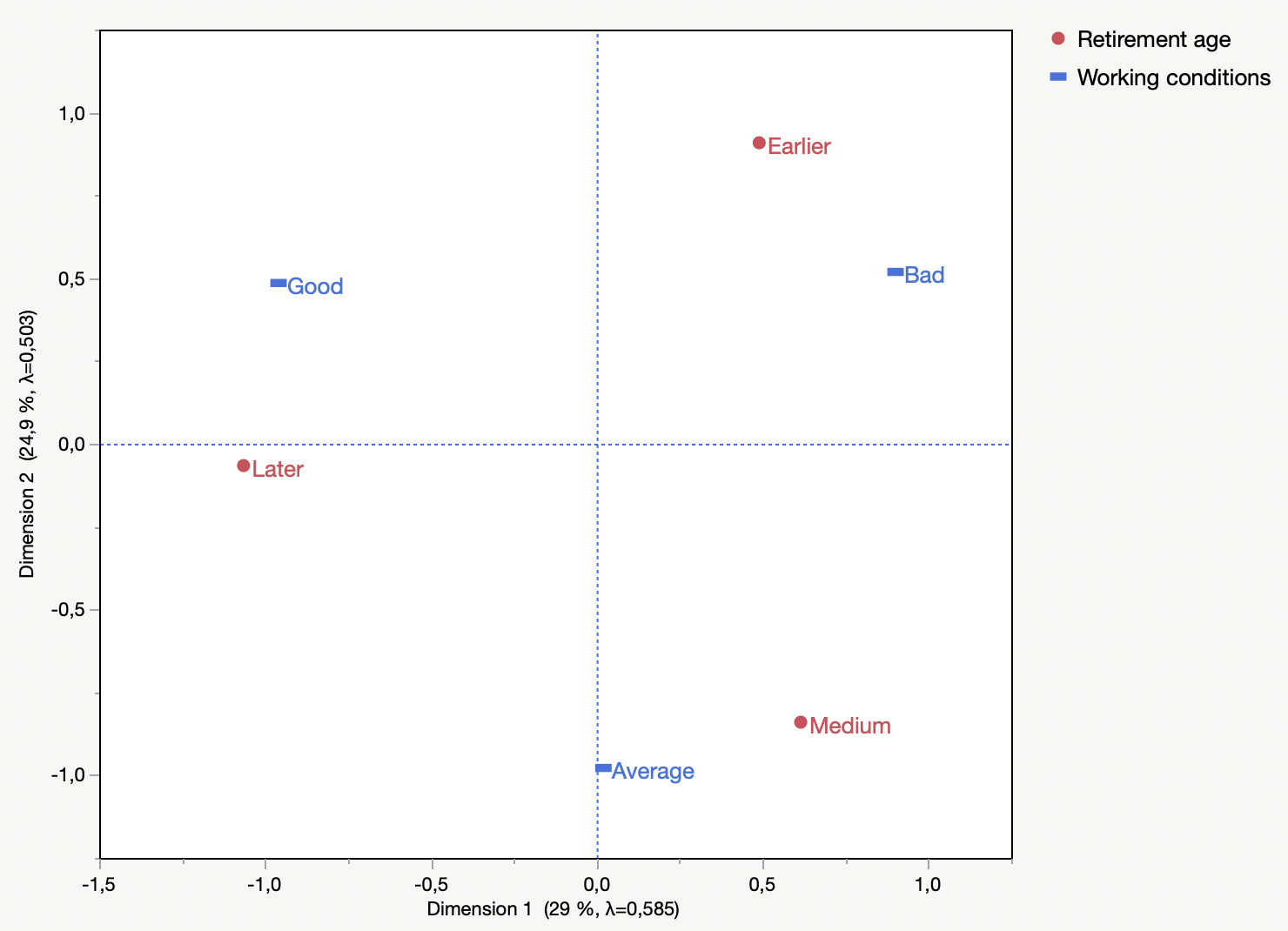
**

**Table S2.** Working conditions, demographics and health status before retirement according to mortality and self-rated health after retirement (n=13,378).

|  | |  |  | **Mortality** | | **Self-rated health** | |
| --- | --- | --- | --- | --- | --- | --- | --- |
|  | |  | **Full Sample** | **Alive** | **Dead** | **Good** | **Suboptimum** |
|  | |  | 13378 | 12276 | 1102 | 7010 | 6368 |
| **Working conditions** | *Good* | | 3983 (29.8%) | 3675 (92.3%) | 308 (7.7%) | 2458 (61.7%) | 1525 (38.3%) |
|  | *Average* | | 4552 (34%) | 4197 (92.2%) | 355 (7.8%) | 2418 (53.1%) | 2134 (46.9%) |
|  | *Bad* | | 4841 (36.2%) | 4403 (91%) | 438 (9%) | 2133 (44.1%) | 2708 (55.9%) |
| **Retirement age** | *Earlier (37 to 52y)* | | 4357 (32.6%) | 3942 (90.5%) | 415 (9.5%) | 2179 (50%) | 2178 (50%) |
|  | *Medium (53 to 54y)* | | 4423 (33.1%) | 4074 (92.1%) | 349 (7.9%) | 2384 (53.9%) | 2039 (46.1%) |
|  | *Later (55 to 60y)* | | 4598 (34.4%) | 4260 (92.6%) | 338 (7.4%) | 2447 (53.2%) | 2151 (46.8%) |
| **Sex** | *Women* | | 2575 (19.2%) | 2448 (95.1%) | 127 (4.9%) | 1291 (50.1%) | 1284 (49.9%) |
|  | *Men* | | 10803 (80.8%) | 9828 (91%) | 975 (9%) | 5719 (52.9%) | 5084 (47.1%) |
| **Birth year** | *1947 to 1954* | | 3803 (28.4%) | 3600 (94.7%) | 203 (5.3%) | 2229 (58.6%) | 1574 (41.4%) |
|  | *1944 to 1946* | | 4025 (30.1%) | 3734 (92.8%) | 291 (7.2%) | 2137 (53.1%) | 1888 (46.9%) |
|  | *1939 to 1943* | | 5550 (41.5%) | 4942 (89%) | 608 (11%) | 2644 (47.6%) | 2906 (52.4%) |
| **Retirement year** | *2001 to 2003* | | 4109 (30.7%) | 3914 (95.3%) | 195 (4.7%) | 2457 (59.8%) | 1652 (40.2%) |
|  | *1999 to 2000* | | 3411 (25.5%) | 3168 (92.9%) | 243 (7.1%) | 1840 (53.9%) | 1571 (46.1%) |
|  | *1991 to 1998* | | 5858 (43.8%) | 5194 (88.7%) | 664 (11.3%) | 2713 (46.3%) | 3145 (53.7%) |
| **Social position** | *High* | | 2938 (22%) | 2730 (92.9%) | 208 (7.1%) | 1732 (59%) | 1206 (41%) |
|  | *Middle* | | 7798 (58.3%) | 7171 (92%) | 627 (8%) | 4076 (52.3%) | 3722 (47.7%) |
|  | *Low* | | 2642 (19.7%) | 2375 (89.9%) | 267 (10.1%) | 1202 (45.5%) | 1440 (54.5%) |
| **Hospitalization^¥^** | *No* | | 11286 (84.4%) | 10364 (91.8%) | 922 (8.2%) | 6102 (54.1%) | 5184 (45.9%) |
|  | *Yes* | | 2092 (15.6%) | 1912 (91.4%) | 180 (8.6%) | 908 (43.4%) | 1184 (56.6%) |
| **Physical illness^¥, µ^** | *No* | | 12310 (92%) | 11362 (92.3%) | 948 (7.7%) | 6619 (53.8%) | 5691 (46.2%) |
|  | *Yes* | | 1068 (8%) | 914 (85.6%) | 154 (14.4%) | 391 (36.6%) | 677 (63.4%) |
| **High sickness absence^¥^** | *No* | | 10294 (76.9%) | 9502 (92.3%) | 792 (7.7%) | 5759 (55.9%) | 4535 (44.1%) |
|  | *Yes* | | 3084 (23.1%) | 2774 (89.9%) | 310 (10.1%) | 1251 (40.6%) | 1833 (59.4%) |
| **Depression^¥^** | *No* | | 11043 (82.5%) | 10134 (91.8%) | 909 (8.2%) | 6295 (57%) | 4748 (43%) |
|  | *Yes* | | 2335 (17.5%) | 2142 (91.7%) | 193 (8.3%) | 715 (30.6%) | 1620 (69.4%) |
| **Musculoskeletal problems^¥^** | *No* | | 6245 (46.7%) | 5703 (91.3%) | 542 (8.7%) | 3738 (59.9%) | 2507 (40.1%) |
|  | *Yes* | | 7133 (53.3%) | 6573 (92.1%) | 560 (7.9%) | 3272 (45.9%) | 3861 (54.1%) |
| **Sleep problems^¥^** | *No* | | 9643 (72.1%) | 8854 (91.8%) | 789 (8.2%) | 5586 (57.9%) | 4057 (42.1%) |
|  | *Yes* | | 3735 (27.9%) | 3422 (91.6%) | 313 (8.4%) | 1424 (38.1%) | 2311 (61.9%) |

**^¥^** Assessed during the 2 years before retirement.

**^µ^** Chronic bronchitis or asthma, angina, myocardial infarction, stroke, diabetes, or cancer.

* p-values are significant (p<0.01).

**Table S3.** Univariate associations of working conditions, demographics and health status before retirement with mortality and suboptimum self-rated health after retirement (n=13,378).

|  | | **Mortality** | **Suboptimum self-rated health** |
| --- | --- | --- | --- |
|  |  | *HR (SE) ^α^ / p-value* | *HR (SE) ^α^ / p-value* |
| **Working conditions** | *Good* | 1.00 | 1.00 |
|  | *Average* | 1.51 (0.16) / 0.0092* | 1.30 (0.04) / <0.0001* |
|  | *Bad* | 1.55 (0.12) / 0.0002* | 1.73 (0.04) / <0.0001* |
| **Retirement age** | *Earlier (37 to 52y)* | 1.00 | 1.00 |
|  | *Medium (53 to 54y)* | 1.11 (0.10) / 0.2916 | 0.94 (0.03) / 0.0469 |
|  | *Later (55 to 60y)* | 0.78 (0.10) / 0.5267 | 0.97 (0.03) / 0.3160 |
| **Sex** | *Women* | 1.00 | 1.00 |
|  | *Men* | 1.85 (0.19) / 0.0009* | 0.88 (0.04) / 0.0010* |
| **Birth year** | *1947 to 1954* | 1.00 | 1.00 |
|  | *1944 to 1946* | 1.11 (0.11) / 0.3203 | 1.13 (0.03) / 0.0005* |
|  | *1939 to 1943* | 1.44 (0.10) / 0.0002* | 1.17 (0.03) / <0.0001* |
| **Retirement year** | *2001 to 2003* | 1.00 | 1.00 |
|  | *1999 to 2000* | 1.16 (0.11) / 0.1500 | 1.07 (0.03) / 0.0377 |
|  | *1991 to 1998* | 1.30 (0.09) / 0.0041* | 1.17 (0.03) / <0.0001* |
| **Social position** | *High* | 1.00 | 1.00 |
|  | *Middle* | 1.23 (0.13) / 0.1094 | 1.18 (0.04) / <0.0001* |
|  | *Low* | 1.33 (0.14) / 0.0448 | 1.41 (0.05) / <0.0001* |
| **Hospitalization^¥^** | *No* | 1.00 | 1.00 |
|  | *Yes* | 1.06 (0.15) / 0.0678 | 1.41 (0.04) / <0.0001* |
| **Physical illness^¥, µ^** | *No* | 1.00 | 1.00 |
|  | *Yes* | 1.87 (0.13) / <0.0001* | 1.75 (0.04) / <0.0001* |
| **High sickness absence^¥^** | *No* | 1.00 | 1.00 |
|  | *Yes* | 1.17 (0.14) / 0.2655 | 1.59 (0.03) / <0.0001* |
| **Depression^¥^** | *No* | 1.00 | 1.00 |
|  | *Yes* | 0.94 (0.11) / 0.5899 | 2.28 (0.03) / <0.0001* |
| **Musculoskeletal problems^¥^** | *No* | 1.00 | 1.00 |
|  | *Yes* | 0.85 (0.12) / 0.1796 | 1.53 (0.03) / <0.0001* |
| **Sleep problems^¥^** | *No* | 1.00 | 1.00 |
|  | *Yes* | 0.90 (0.13) / 0.4340 | 1.78 (0.03) / <0.0001* |

^α^ Hazard ratios (HR) and their standard errors (SE) were estimated using log-rank tests.

**^¥^** Assessed during the 2 years before retirement.

**^µ^** Chronic bronchitis or asthma, angina, myocardial infarction, stroke, diabetes, or cancer.

* p-values are significant (p<0.01).

**Fig S4.** Average silhouette coefficient distribution for each cluster analysis model according to the number of clusters.

**
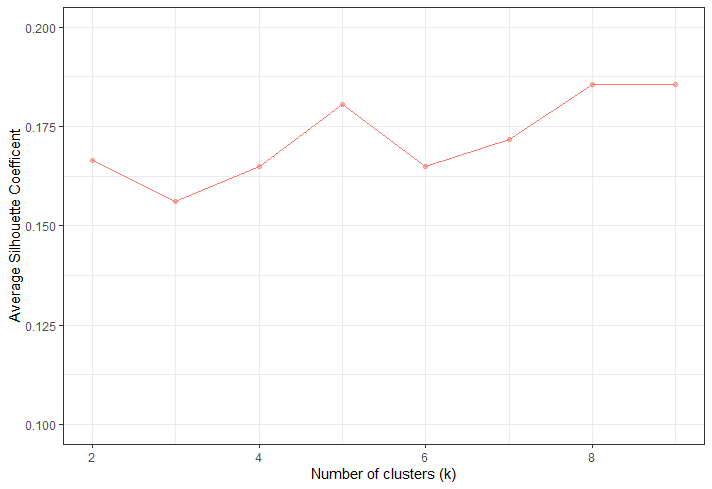
**

**Table S4.** Multivariable weighted Cox regression models examining the associations of working conditions, retirement age, demographics, health status, smoking, leisure-time physical inactivity and non-moderate alcohol consumption before retirement with mortality and suboptimum self-rated health after retirement (n=2,017).

|  | | **Mortality** | **Suboptimum self-rated health** |
| --- | --- | --- | --- |
|  |  | *HR (SE) ^α^ / p-value* | *HR (SE) ^α^ / p-value* |
| **Working conditions** | *Good* | 1.00 | 1.00 |
|  | *Average* | 0.82 (0.30) / 0.5046 | 1.05 (0.10) / 0.6436 |
|  | *Bad* | 1.90 (0.30) / 0.0329* | 1.30 (0.10) / 0.0127* |
| **Retirement age** | *Earlier (37 to 52y)* | 1.00 | 1.00 |
|  | *Medium (53 to 54y)* | 0.89 (0.28) / 0.6670 | 0.92 (0.09) / 0.3553 |
|  | *Later (55 to 60y)* | 0.70 (0.31) / 0.2588 | 0.87 (0.11) / 0.1736 |
| **Sex** | *Women* | 1.00 | 1.00 |
|  | *Men* | 1.69 (0.35) / 0.1274 | 0.86 (0.11) / 0.1636 |
| **Birth year** | *1947 to 1954* | 1.00 | 1.00 |
|  | *1944 to 1946* | 1.24 (0.37) / 0.5530 | 1.14 (0.11) / 0.2330 |
|  | *1939 to 1943* | 1.85 (0.46) / 0.1842 | 1.34 (0.15) / 0.0436* |
| **Retirement year** | *2001 to 2003* | 1.00 | 1.00 |
|  | *1999 to 2000* | 1.15 (0.36) / 0.6956 | 0.91 (0.11) / 0.3530 |
|  | *1991 to 1998* | 0.96 (0.44) / 0.9192 | 0.82 (0.13) / 0.1269 |
| **Social position** | *High* | 1.00 | 1.00 |
|  | *Middle* | 1.85 (0.29) / 0.0341* | 1.11 (0.10) / 0.2733 |
|  | *Low* | 1.16 (0.38) / 0.6915 | 1.32 (0.12) / 0.0183* |
| **Hospitalization^¥^** | *No* | 1.00 | 1.00 |
|  | *Yes* | 1.42 (0.28) / 0.2195 | 1.07 (0.10) / 0.5377 |
| **Physical illness^¥, µ^** | *No* | 1.00 | 1.00 |
|  | *Yes* | 1.29 (0.34) / 0.4597 | 1.74 (0.12) / <0.0001* |
| **High sickness absence^¥^** | *No* | 1.00 | 1.00 |
|  | *Yes* | 0.77 (0.29) / 0.3614 | 1.19 (0.09) / 0.0624 |
| **Depression^¥^** | *No* | 1.00 | 1.00 |
|  | *Yes* | 1.18 (0.38) / 0.6557 | 1.81 (0.09) / <0.0001* |
| **Musculoskeletal problems^¥^** | *No* | 1.00 | 1.00 |
|  | *Yes* | 0.78 (0.24) / 0.3119 | 1.41 (0.08) / <0.0001* |
| **Sleep problems^¥^** | *No* | 1.00 | 1.00 |
|  | *Yes* | 1.06 (0.25) / 0.8113 | 1.33 (0.09) / 0.0009* |
| **Smoking^¥^** | *No* | 1.00 | 1.00 |
|  | *Yes* | 0.93 (0.60) / 0.8995 | 0.72 (0.31) / 0.2959 |
| **Non-moderate alcohol consumption^¥^** | *No* | 1.00 | 1.00 |
|  | *Yes* | 1.13 (0.31) / 0.6922 | 1.12 (0.12) / 0.3116 |
| **Leisure-time physical inactivity^¥^** | *No* | 1.00 | 1.00 |
|  | *Yes* | 0.97 (0.56) / 0.9624 | 1.20 (0.15) / 0.2301 |

^α^ Hazard ratios (HR) and their standard errors (SE) were estimated using multivariable weighted Cox regression models.

**^¥^** Assessed during the 2 years before retirement.

**^µ^** Chronic bronchitis or asthma, angina, myocardial infarction, stroke, diabetes, or cancer.

* p-values are significant (p<0.05).

**Table S5.** Univariate Cox proportional hazard regression models examining the associations of clusters of workers with mortality and suboptimum self-rated health after retirement, while excluding outliers^¥^.

|  | **Mortality** | | **Suboptimum self-rated health** | |
| --- | --- | --- | --- | --- |
| **Cluster** | *HR (SE) ^α^ / p-value* | *E-value  (lower estimate)* | *HR (SE)* ^β^ */ p-value* | *E-value  (lower estimate)* |
| 4 | 1.00 |  | 1.00 |  |
| 1 | 0.82 (0.13) / 0.1289 | - | 1.09 (0.05) / 0.0927 | - |
| 2 | 1.39 (0.12) / 0.0077* | 1.82 (1.32) | 2.03 (0.06) / <0.0001* | 2.65 (2.40) |
| 3 | 0.93 (0.17) / 0.6596 | - | 1.04 (0.07) / 0.6268 | - |
| 5 | 0.99 (0.12) / 0.9365 | - | 1.17 (0.05) / 0.0031* | 1.48 (1.24) |
| 6 | 0.92 (0.13) / 0.4946 | - | 1.23 (0.05) / <0.0001* | 1.58 (1.36) |
| 7 | 1.29 (0.13) / 0.0459 | - | 1.47 (0.06) / <0.0001* | 1.94 (1.72) |
| 8 | 0.97 (0.16) / 0.8567 | - | 1.25 (0.07) / 0.0006* | 1.61 (1.34) |

Cluster 1: younger workers who retired between 2001 and 2003; Cluster 2: older workers with bad working conditions who retired between 1991 and 1998; Cluster 3: younger retired workers with high social position; Cluster 4: older retired workers with high social position; Cluster 5: older retired workers with middle social position; Cluster 6: younger retired workers with middle social position; Cluster 7: retired workers with low social position; Cluster 8: younger workers who retired between 1999 and 2000.

^α^ Hazard ratios (HR) and their standard errors (SE) were estimated using Cox proportional hazard regression models. Proportional hazard assumption χ²=8.58 (df=7; p=0.28).

^β^ Hazard ratios (HR) and their standard errors (SE) were estimated using Cox Proportional hazard regression models. Proportional hazard assumption χ²=11.7 (df=7; p=0.11).

E-values were used to quantify sensitivity to unmeasured confounders.

^¥^ Outliers were defined as observations that were more than 2 interquartile ranges below the first quartile or above the third quartile, based on Martingale-based residuals.

* p-values are significant (p<0.01).

**Table S6.** Multivariate Cox proportional hazard regression models examining the associations of clusters of workers with mortality and suboptimum self-rated health after retirement, adjusted for smoking, leisure-time physical inactivity and non-moderate alcohol consumption (n=2,017).

|  | **Mortality** | **Suboptimum self-rated health** |
| --- | --- | --- |
| **Cluster** | *HR (SE) ^α^ / p-value* | *HR (SE) ^α^ / p-value* |
| 4 | 1.00 | 1.00 |
| 1 | 0.92 (0.40) / 0.8272 | 1.05 (0.13) / 0.7302 |
| 2 | 2.04 (0.34) / 0.0363* | 1.32 (0.14) / 0.0455* |
| 3 | 0.80 (0.53) / 0.6730 | 1.03 (0.17) / 0.8453 |
| 5 | 1.15 (0.35) / 0.6975 | 1.01 (0.14) / 0.9241 |
| 6 | 1.07 (0.37) / 0.8513 | 1.13 (0.13) / 0.3474 |
| 7 | 1.27 (0.39) / 0.5387 | 1.33 (0.14) / 0.0513 |
| 8 | 0.78 (0.54) / 0.6458 | 1.17 (0.17) / 0.3627 |

Cluster 1: younger workers who retired between 2001 and 2003; Cluster 2: older workers with bad working conditions who retired between 1991 and 1998; Cluster 3: younger retired workers with high social position; Cluster 4: older retired workers with high social position; Cluster 5: older retired workers with middle social position; Cluster 6: younger retired workers with middle social position; Cluster 7: retired workers with low social position; Cluster 8: younger workers who retired between 1999 and 2000.

* p-values are significant (p<0.01).

**Table S7.** Univariate weighted Cox regression models examining the associations of retirement age with mortality and suboptimum self-rated health after retirement in each cluster of workers.

|  | ***Retirement age*** | **Cluster 1** | **Cluster 2** | **Cluster 3** | **Cluster 4** | **Cluster 5** | **Cluster 6** | **Cluster 7** |
| --- | --- | --- | --- | --- | --- | --- | --- | --- |
|  |  | *HR (SE) ^α^ / p-value* | *HR (SE) ^α^ / p-value* | *HR (SE) ^α^ / p-value* | *HR (SE) ^α^ / p-value* | *HR (SE) ^α^ / p-value* | *HR (SE) ^α^ / p-value* | *HR (SE) ^α^ / p-value* |
| **Mortality** | *Earlier*  *(37 to 52y)* | 1.00 | 1.00 | 1.00 | 1.00 | 1.00 | 1.00 | 1.00 |
|  | *Medium*  *(53 to 54y)* | 0.97 (0.20) / 0.8725 | 0.92 (0.17) / 0.6104 | 0.63 (0.34) / 0.1796 | 0.87 (0.42) / 0.7374 | 0.97 (0.19) / 0.8925 | 0.70 (0.17) / 0.0428 | 1.23 (0.20) / 0.3907 |
|  | *Later*  *(55 to 60y)* | 1.10 (0.24) / 0.6912 | 0.64 (0.19) / 0.0168 | 0.37 (0.33) / 0.0022 | 0.76 (0.34) / 0.3934 | 1.01 (0.17) / 0.9401 | 0.82 (0.20) / 0.3219 | 1.01 (0.23) / 0.9415 |
| **Suboptimum self-rated health** | *Earlier*  *(37 to 52y)* | 1.00 | 1.00 | 1.00 | 1.00 | 1.00 | 1.00 | 1.00 |
|  | *Medium*  *(53 to 54y)* | 0.93 (0.07) / 0.2785 | 1.02 (0.08) / 0.7706 | 0.85 (0.18) / 0.3856 | 0.86 (0.17) / 0.3783 | 0.89 (0.08) / 0.1627 | 0.93 (0.07) / 0.3224 | 0.99 (0.09) / 0.9279 |
|  | *Later*  *(55 to 60y)* | 1.01 (0.08) / 0.9091 | 0.87 (0.08) / 0.0817 | 0.84 (0.16) / 0.2977 | 0.93 (0.14) / 0.6116 | 1.05 (0.08) / 0.5373 | 1.03 (0.08) / 0.6711 | 1.04 (0.08) / 0.6158 |

Cluster 1: younger workers who retired between 2001 and 2003; Cluster 2: older workers with bad working conditions who retired between 1991 and 1998; Cluster 3: younger retired workers with high social position; Cluster 4: older retired workers with high social position; Cluster 5: older retired workers with middle social position; Cluster 6: younger retired workers with middle social position; Cluster 7: retired workers with low social position. Note that the cluster 8 is not represented as it does not present all retirement age terciles.

* p-values are significant (p<0.01).

**Table S8.** Multivariate Cox regression models examining the interaction between clusters of workers and retirement age with mortality and suboptimum self-rated health after retirement, adjusted for smoking, leisure-time physical inactivity and non-moderate alcohol consumption (n=13,378).

|  | | **Mortality** | **Suboptimum self-rated health** |
| --- | --- | --- | --- |
|  |  | *HR (SE) ^α^ / p-value* | *HR (SE) ^β^ / p-value* |
| **Cluster** | *Cluster 4* | 1.00 | 1.00 |
|  | *Cluster 1* | 0.82 (0.29) / 0.4849 | 1.10 (0.13) / 0.4737 |
|  | *Cluster 2* | 1.66 (0.27) / 0.0627 | 1.77 (0.14) / <0.0001* |
|  | *Cluster 3* | 1.25 (0.37) / 0.5467 | 1.19 (0.18) / 0.3435 |
|  | *Cluster 5* | 1.04 (0.28) / 0.8843 | 1.20 (0.14) / 0.1929 |
|  | *Cluster 6* | 1.07 (0.28) / 0.8097 | 1.25 (0.13) / 0.0986 |
|  | *Cluster 7* | 1.35 (0.28) / 0.2875 | 1.40 (0.14) / 0.0154 |
|  | *Cluster 8* | 1.00 (0.28) / 0.9897 | 1.24 (0.13) / 0.1037 |
| **Retirement age** | *Earlier (37 to 52y)* | 1.00 | 1.00 |
|  | *Medium (53 to 54y)* | 1.12 (0.33) / 0.7273 | 0.93 (0.16) / 0.6406 |
|  | *Later (55 to 60y)* | 1.02 (0.28) / 0.9495 | 1.01 (0.14) / 0.9475 |
| **Interaction** | *Cluster 1*Medium* | 0.89 (0.38) / 0.7595 | 1.02 (0.18) / 0.9158 |
|  | *Cluster 2*Medium* | 0.81 (0.37) / 0.5624 | 1.09 (0.18) / 0.6505 |
|  | *Cluster 3*Medium* | 0.87 (0.48) / 0.7702 | 0.88 (0.23) / 0.5792 |
|  | *Cluster 5*Medium* | 0.81 (0.38) / 0.5881 | 0.95 (0.18) / 0.7763 |
|  | *Cluster 6*Medium* | 0.70 (0.38) / 0.3476 | 1.00 (0.18) / 0.9842 |
|  | *Cluster 7*Medium* | 0.89 (0.38) / 0.7580 | 1.09 (0.19) / 0.6541 |
|  | *Cluster 8*Medium* | 1.23 (0.68) / 0.7574 | 0.94 (0.32) / 0.8503 |
|  | *Cluster 1*Later* | 1.18 (0.37) / 0.6479 | 1.04 (0.16) / 0.8092 |
|  | *Cluster 2*Later* | 0.68 (0.33) / 0.2431 | 0.86 (0.16) / 0.3298 |
|  | *Cluster 3*Later* | 0.53 (0.46) / 0.1689 | 0.85 (0.20) / 0.4070 |
|  | *Cluster 5*Later* | 1.00 (0.33) / 0.9897 | 1.02 (0.16) / 0.8742 |
|  | *Cluster 6*Later* | 0.88 (0.34) / 0.7200 | 1.03 (0.16) / 0.8666 |
|  | *Cluster 7*Later* | 0.94 (0.34) / 0.8637 | 1.10 (0.16) / 0.5440 |
|  | *Cluster 8*Later* | N/A | N/A |

Cluster 1: younger workers who retired between 2001 and 2003; Cluster 2: older workers with bad working conditions who retired between 1991 and 1998; Cluster 3: younger retired workers with high social position; Cluster 4: older retired workers with high social position; Cluster 5: older retired workers with middle social position; Cluster 6: younger retired workers with middle social position; Cluster 7: retired workers with low social position; Cluster 8: younger workers who retired between 1999 and 2000.

^α^ Hazard ratios (HR) and their standard errors (SE) were estimated using Cox proportional hazard regression models. Proportional hazard assumption χ²=17.64 (df=22; p=0.73).

*^β^* Hazard ratios (HR) and their standard errors (SE) were estimated using Cox proportional hazard regression models. Proportional hazard assumption χ²=36.95 (df=22; p=0.02).

* p-values are significant (p<0.01). N/A: not applicable.
